# Monitoring Lead Concentration in the Surrounding Environmental Components of a Lead Battery Company: Plants, Air and Effluents—Case Study, Kenya

**DOI:** 10.3390/ijerph19095195

**Published:** 2022-04-25

**Authors:** Jeremiah Otieno, Przemysław Kowal, Jacek Mąkinia

**Affiliations:** Faculty of Civil and Environmental Engineering, Gdansk University of Technology, Narutowicza 11/12, 80-233 Gdansk, Poland; jeremiah.otieno@pg.edu.pl (J.O.); jmakinia@pg.edu.pl (J.M.)

**Keywords:** developing countries, lead pollution, lead–acid battery, heavy metals

## Abstract

Lead (Pb) pollution from smelters and lead–acid battery has become a serious problem worldwide owing to its toxic nature as a heavy metal. Stricter regulations and monitoring strategies have been formulated, legislated and implemented in various parts of the world on heavy metal usage. Developed countries such as the USA and in Europe largely operate within the set standards, however, developing countries such as Kenya, Nigeria and India, with limited regulatory capacity, resources and sufficient data, face poor Pb waste management and exposure of the population to health risks. This study assessed the pollution concerns from Associated Battery Manufacturers (East Africa) Limited (ABM), located in the Nairobi Industrial Area in Kenya. Samples of air, extracts from plants (leaves) and factory wastewaters were taken from different operations units, prepared and analysed with Atomic Absorption Spectrometry (AAS). Pb traces remained fairly controlled with averages of 1.24 ± 0.42 parts per million (ppm), 1.21 ± 0.02 ppm and 0.29 ± 0.01 ppm in the air, plant extracts and effluents, respectively. The conducted research shows that the obtained lead concentrations in the air, wastewater and surrounding plants exceeded the recommended standards, and are potentially harmful not only to workers, but also to the surrounding villages.

## 1. Introduction

The rapid shift towards producing and using clean energy to replace fossil fuels has increased the demand for batteries. Among the available batteries, lithium ion and lead (Pb)–acid batteries have the dominant market share. Lead–acid battery factories and smelters rely heavily on lead ores as the main input raw material, with lead content of more than 70% wet weight. 

As with other heavy metals, lead is highly toxic and persistent in the environment, thus the processing of lead ores, including the batteries’ production, generates high environmental and health risks [1]. For instance, lead affects nerve cells in the spinal cord, disrupts the functioning of the reproductive system and has been identified as a neurotoxicant involved in the etiology of mental disorders [2]. Moreover, children, unlike adults, are disproportionately affected when exposed to lead above 10 µg/dL in their blood, with potential effects of learning impairment and behavioural problems, and at very high levels, seizures, coma or even death [3]. According to the US Centers for Disease Control and Prevention (CDC) and the World Health Organization (WHO), a blood lead level of 10 μg/dL or above is a cause for concern, with the latest revision at 5 μg/dL [4,5].

While there seems to be general consensus to limit and discontinue the use of lead-related products, including lead-based fuels and paints, the effectiveness of these coordinated efforts is strictly dependent on the law regulations and contamination monitoring programmes implemented by responsible authorities. In developed countries and regions, such as European countries, Canada and the United States, the use of lead is limited or prohibited and the effects of its application are monitored to ensure a sustainable impact on the environment. However, lead is still heavily used in developing countries due to a combination of different social, economic and political factors [6].

The use of lead in developing regions, i.e., in Africa, is characterised by a lack of strong monitoring systems, inadequate resources and technology, insufficient scientific data for decision making and sometimes incorrect management by responsible authorities, making the environment and working areas vulnerable to adversities from lead pollutant sources [1]. In particular, workers in certain industrial branches related to the use of lead, such as battery manufacturing, are considered to be the most vulnerable risk group for lead poisoning. 

A number of unit processes in lead battery factories handle lead in a variety of forms. For instance, mixing and pasting unit processes require powdered lead as one of the ingredients for plate formation and paste generation. Additionally, brushing the plates on the assembly lines produces tiny lead particles that fill the air with fine invisible droplets, potentially transporting to the respiratory tract, and some accumulating on nearby soil and plants. The factory also has filters that are used to extract lead dust and, when these filters are cleaned, a lot of lead-loaded dust enters the air. As well as air contamination, dust washed from the factory floor, especially in the formation and pasting unit processes, generates wastewater loaded with lead.

Some preliminary examination of battery factory workers in Nnewi (Nigeria) recorded elevated Pb levels of more than 5 μg/dL in the blood compared to regular individuals, resulting in heavy metal toxicity and depletion in some vital micronutrients [7,8]. 

In the case of Kenya, lead poisoning from the metal refinery (EPZ) of the Owino Uhuru community has been well documented in recent history. The operations of the metal refinery (EPZ) without strict regulations and monitoring resulted in poor waste management and disposal, characterised by lead-filled wastewater from factory hall floors that entered the open flow of streets. Moreover, lead-containing vapours and lumpy sediments that showed a tendency to settle on soils and plants were transferred into the air. In consequence, more than 3000 inhabitants have been exposed to elevated lead levels in various environmental components, resulting in traceable lead-related deaths of 20 people since the facility was opened, according to the Centre for Justice, Governance and Environment [9,10].

The presented examples of lead poisoning in developing countries underline the responsibility of companies, that deal with lead in their operations, to investigate and control lead levels in the working areas and surroundings to offer monitoring for data-driven decisions and improving working conditions.

This research paper assesses the degree of lead contamination in the work areas around a lead battery manufacturing plant, Associated Battery Manufacturing (East Africa) Limited (ABM) located in an industrial area of Nairobi, Kenya. Particular emphasis was placed on the measurements of lead concentrations from factory wastewater, lead in the air in different sections of the operations and lead accumulation in the nearby tropical plants. The key aspect of the conducted experiments was to assess the degree of exposure of facility employees to lead in the air and lead accumulation in selected work zones. In addition, monitoring data were obtained to verify whether the lead concentrations directed to the sewage system meet the recommended safe threshold.

## 2. Materials and Methods

### 2.1. Plant Location and Setting 

**Figure 1:**
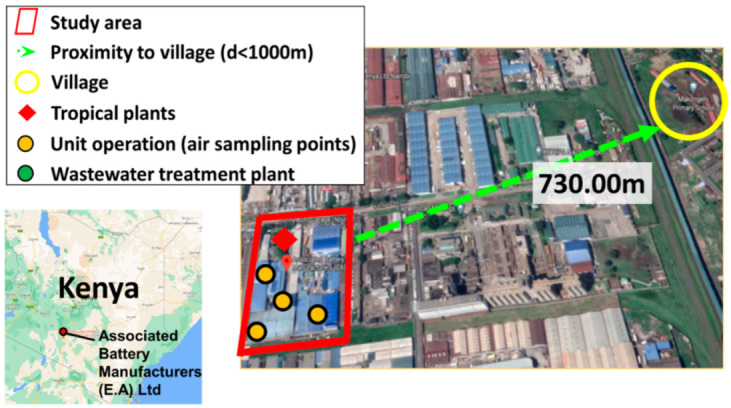
Research area regarding proximity to villages of Associated Battery Manufacturing and location of lead sampling points, Associated Battery Manufacturing (East Africa) Limited, Nairobi, Kenya.

Associated Battery Manufacturing (ABM) is located in Nairobi, Kenya (−1.3052, 36.8537), in an industrial district 900 m from nearby communities and operates on a continuous production system (Figure 1). In order to validate lead contamination of the work zones and the area surrounding the factory, samples of air, effluent wastewater and plants were collected. Sampling points have been designated in different sections of the factory. 

**Figure 2:**
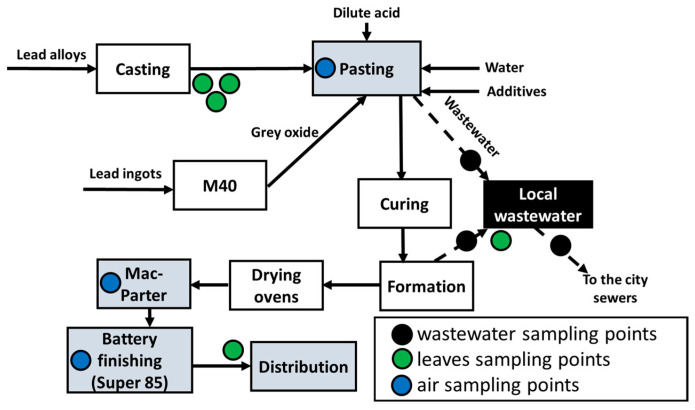
Schematic mapping of battery production stages and unit operations with marked points for air sampling, leaf sampling and wastewater quality measurements.

For lead measurements in the air, sampling points were placed in different sections with the highest probability of lead dust formation: casting and paste generation (Pasting section), paste mixing and plate preparation (Mac-Parter section) and battery finishing section (Super 85) as shown in Figure 2. Subsequent assessment of the precautionary measures taken to prevent lead dust transfer into the nearby villages of Makongeni, Mukuru and Mamboleo was carried out. 

The forming and pasting sections were selected for monitoring lead concentrations, which contributed to the generation of wastewater directed to the locally installed wastewater treatment plant. Additionally, samples of treated wastewater were collected to determine effectiveness of the local wastewater treatment system.

Another marker of lead contamination was leaf samples of tropical plants growing on the factory premises. It was assumed that the lead concentrations in the plant leaves were the sum of the surface lead deposits from the air and those possibly absorbed by the roots.

### 2.2. Sampling and Analyses

#### 2.2.1. Air and Dust Samples

The sections with the highest potential for lead dust release into the air were selected for sampling (Pasting, Mac-Parter, Super 85). Within each section, two measurement points were randomly selected. Sampling of air and dust at each measuring point was performed periodically for 8 h on each working day over a period of 7 days (Table S1).

Air sampling was performed by a Casella Cel analytical tool according to the manufacturer’s protocol. Dust in the air rich in lead particles was deposited on the filter paper mounted with Casella Cel and the level of air pollution was determined based on the sediment analysis. The cartridge of the device was weighed first separately and later against the dried filter paper. A lead sampling cassette was assembled with filter paper between the support and the cover. A flexible tube was used to connect the sampling head and the pump. After the measurement, the filter paper was reweighed together with the cartridge, then digested with 1:3 solution of perchloric (HClO_4_) and nitric (HNO_3_) acids for 15 min. Prepared samples were run with Atomic Absorption Spectrophotometry (AAS) with blanks at a wavelength of 283.3 nm against standard lead solutions (5 ppm and 10 ppm). Calculations of the lead in the air in mg/m^3^ were carried out with Equation (1).

Lead in air = lead conc. (ppm) × 50/volume of air (liters)(1)

where dust level (ppm) = 106 × weight of dust (g)/volume of air (liters) and weight of dust = final filter weight (WtB) − initial filter weight (WtA).

#### 2.2.2. Plant Leaf Extract

Sampling of plant extracts consisted of careful picking of leaves of plants growing within selected production sections (pasting, local wastewater treatment plant and distribution). The sampling was carried out over 5 working days with each day dedicated to a different section (Table S3). The plant species from which the leaves were harvested was a typical “tropical plant” specific to the Nairobi region of Kenya. Two samples (20 leaves per sample) were taken from each plant in the sampling sections at the end of an 8 h work shift.

Preparation of test plant samples consisted of drying plant leaves in an oven overnight at 60 °C. The dried samples were then milled through a 0.5 mm sieve without prior washing. Subsequently, 1 g of the dried sample was weighed, transferred to a glass beaker and digested with 20 mL of HClO_4_ in a hot sand bath to enhance digestion. The end of complete digestion was assumed when the brown NO_2_ vapours were stripped and a colourless solution was obtained. Then, 20 mL of distilled water was added to the sample and allowed to cool to room temperature. The plant extract was filtrated through Whatman filter paper into a 100 mL volumetric flask and filled to the mark with distilled water. Filtrated plant extracts were then analysed for the lead concentration by flame Atomic Absorption Spectrophotometry (AAS). A blank sample analysis was incorporated for each run and the average of the lead concentration from the two independent replicates was calculated.

#### 2.2.3. Wastewater

Two sets of effluent wastewater samples, one taken before and one after the wastewater treatment process, were collected within the local wastewater treatment plant once a day. Sampling time was within 5 working days and a unified sampling procedure was used. Each day, a mixture of raw wastewater from the pasting and formation units, as well as treated wastewater samples, was collected into a 1 L bottle, filtered in order to remove suspended solids and run in AAS. Notably, Associated Battery Manufacturer (ABM) used soda ash (NaCO_3_) to precipitate lead from wastewater, achieving a low lead concentration when recycling is not required at neutral pH 7.

### 2.3. Analytical Methods

#### Atomic Absorption Spectrophotometry (AAS)

Prepared samples of the air/dust, plant leaf extract and wastewater were analysed with the flame atomic absorption method. Air/dust samples’ absorbance values were compared against the standard lead solutions of 0, 5, 10 and 15 ppm. For the wastewater samples, lead solution standards of 0, 4, 6 and 10 ppm were applied. Plant extracts were run with the same calibration curves as the wastewater samples.

## 3. Results and Discussion

### 3.1. Lead Concentration Monitoring

#### 3.1.1. Lead in the Air/Dust

The highest lead concentrations in the air/dust, above 1.00 ppm, were recorded in the battery assembling sections and subsections including Mac-Parter (subsections A and B −1.81 and 1.43 ppm, respectively) and Super 85 (subsections A and B at 1.23 and 1.03 ppm, respectively) (see supplementary Table S1). The dust from the battery plate preparation stage is clearly a concern to the shift workers in these particular sections, which are the greatest contributors of lead release into the air. This is due to the fact that brushing of plate lags and casing processes for dried paste generate dust particles in the air. The protective infrastructure components are lower fume chambers and dust collectors lowered from the roof top to the point of generation, which sometimes are not efficiently maintained, resulting in leakages. 

The observed lead concentrations in the Mac-Parter and Super 85 sections, in accordance with the literature data, were higher in relation to the concentrations potentially affecting human behaviour. Stretesky and Lynch [11] have observed a positive correlation between crime rates (about four times higher) in countries with air lead levels equivalent to 0.17 µg/m^3^ (170 ppm) in relation to countries with air lead levels equivalent to 0.0 µg/m^3^. However, long-term exposure has to be taken into account. For instance, acute lead intoxication in female workers and cleaners working in a battery-producing factory was diagnosed, with malaise and abdomen-associated pains, after two weeks’ exposure to lead-contaminated air [12]. 

In the pasting section, the measured lead concentrations in the air were lower in relation to the Mac-Parter and battery finishing sections, and were in the range of 0.01 up to 0.70 ppm. Mac-Parter and battery finishing sections are characterised by brushing and griding of the plates, producing a lot of lead dust in the air despite the installed air extractors. A probable explanation for the observed phenomena was higher air moisture which favoured lead deposition in the pasting section. 

#### 3.1.2. Lead in Effluents

Analysis of the raw effluent wastewater samples from the formation and pasting sections revealed stable lead concentrations during the experimental period at the level 0.321 ± 0.004 ppm. Lead removal efficiency at the local WWTP within the factory was low at 9.89% ± 1.32% and the final concentration was 0.289 ± 0.002 ppm (Supplementary Table S2).

Lead in the effluents comes from various unit operation sections, where the major contributors are: paste mixing, curing and formation. The treatment process provided by the addition of NaCO_3_ has been found ineffective and did not meet local requirements regarding lead concentration in the wastewater of <0.01 ppm [13], as presented in Table 1. The WHO standards of 0.01 ppm effluents and the National Environmental Management Authority (NEMA) of Kenya limits of 0.3 ppm are not met at ABM. 

**Figure 3:**
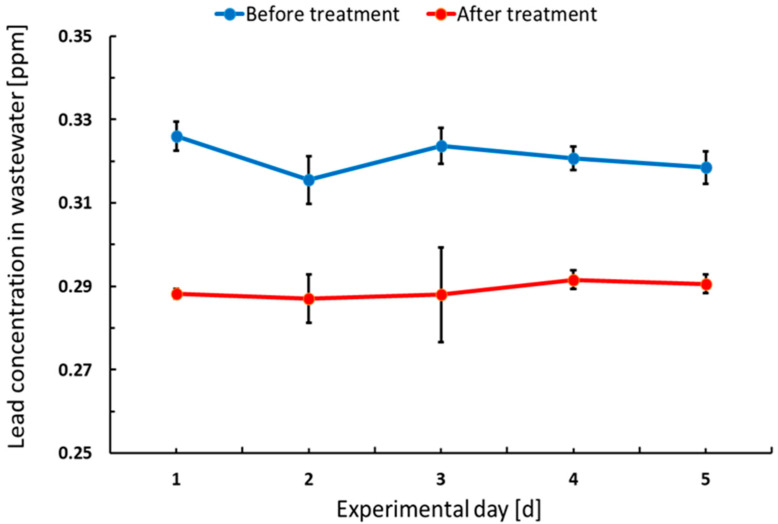
Mean variation in lead concentration in effluent over 5 days from the measurements of factory operations before treatment and after NaCO_3_ treatment in ABM.

Figure 3 shows the variation in lead concentration in the effluent wastewater before and after treatment at the local WTTP by addition of NaCO_3_. Based on performed measurements, NaCO_3_ dosage has to be optimised or new treatment technology has to be proposed to avoid lead particles being transferred into effluent from the sections of the unit operation.

Effluent discharge from batteries is a major contributor of hazardous waste into industrial areas with direct impacts on soil, water and sanitation. 

#### 3.1.3. Lead in Plant Extract

Pb concentrations in the extracts from the leaves of the surrounding tropical plants ranged from 1.18 ppm to 1.23 ppm with an average of 1.21 ± 0.02 ppm (Supplementary Table S3). The limits of the World Health Organization (WHO) and Kenya (NEMA and KEBS) on lead concentrations are ≤0.3 ppm (Table 1). These values are actually higher and pose environmental risk to the communities exposed and are likely to produce elevated Blood Lead Levels (BLLs).

Extracts from plant leaves in the locations next to the wastewater tunnels showed the highest concentrations of up to 1.23 ppm, representing a 4% drop in Pb concentration compared to the plants located 20 m away in the distribution section. The share of lead concentration in plant extracts is a combination of deposits and lead absorbed by the roots. The accumulation of lead in a plant’s tissues is an indication that it is absorbing lead through its roots from nearby soils. Usman et al. [14] observed that increased lead in soils stimulates growth and it accumulates in high concentrations in the plant species *Tetraena qataranse*. *T. qataranse*, which thrives well in arid conditions and is both drought and salt tolerant, demonstrated good Pb accumulation capacity. Our tropical plant extract samples bear similarity in terms of growth conditions. The ability to cope with high levels of Pb occurs via the antioxidant system by synthesizing and increasing the activities of critical enzymes. Sharma and Dubey [15] point out to that despite lead not being an essential element for plants, it is easily absorbed and accumulated in different plant parts from the soils. Uptake of Pb in plants is regulated by pH, particle size and cation exchange capacity of the soils, root exudation and other physical–chemical parameters. Excess Pb causes a number of toxicity symptoms in plants, e.g., stunted growth, chlorosis and blackening of the root system. Furthermore, Pb inhibits photosynthesis, upsets mineral nutrition and water balance, changes hormonal status and affects membrane structure and permeability [16].

Ericson et al. [9] found no statistically significant association between proximity to the lead smelter and soil lead concentrations (*p* < 0.05) in a radius of at least 130 m. This is despite Ericson et al. [9] finding higher lead levels in the plant extracts closer to the factory as a result of elevated soil lead levels in nearby soils. Lead concentration in soils decreases with an increase in distance from the factory.

Additionally, Pb levels in plant extracts are a result of lead deposits on the surfaces of leaves and preserved by careful sampling and digesting the leaf during the sample preparation. Plants growing in areas near casting and distribution had physically dusty surfaces compared to plant leaves in areas around the local wastewater treatment plant. Accumulation of dust particles on the surface of leaves was a result of active lead-filled air particles generated from the unit operations in these sections of the factory. A study on plants 20 m from a lead battery factory suggested that lead particles in air from the company operations are deposited on their surfaces [17,18].

**Figure 4:**
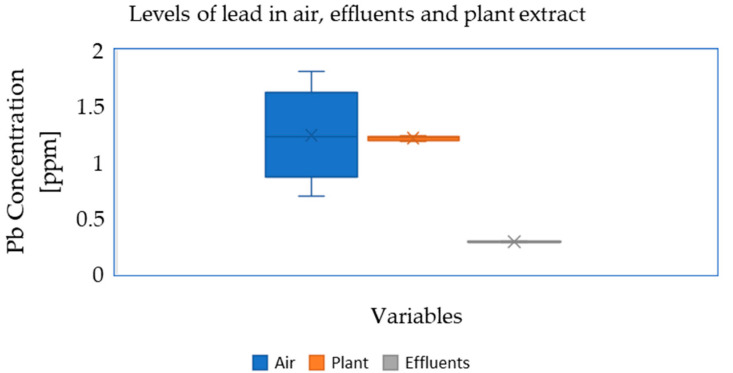
Average variation in Pb concentration in the air, plant leaf extract and effluent wastewater in ppm within Associated Battery Manufacturing (East Africa).

In summary, there were considerably higher lead concentrations in the surrounding plants (soils) and air samples in relation to the effluent wastewater (Figure 4).

The conducted research shows that the obtained lead extracts from the air, wastewater and surrounding plants exceeded the recommended standards, and are potentially harmful not only to workers, but also to the surrounding villages. From this finding, it is necessary to implement permanent monitoring procedures for individual environmental components within the plant, as well as to extend it to neighboring housing estates. Although wastewater is diverted to an external treatment system, internal systems should be checked regularly against established standards. Remarkably, the data revealed that the levels of lead (heavy metal) in the surroundings of Associated Battery Manufacturing deviate from the permissible range and tests and regular measurements should be carried out at similar plants in developing countries. 

### 3.2. Social Impact of Lead Pollution in the Surrounding Environment in Developing Countries

**Figure 5:**
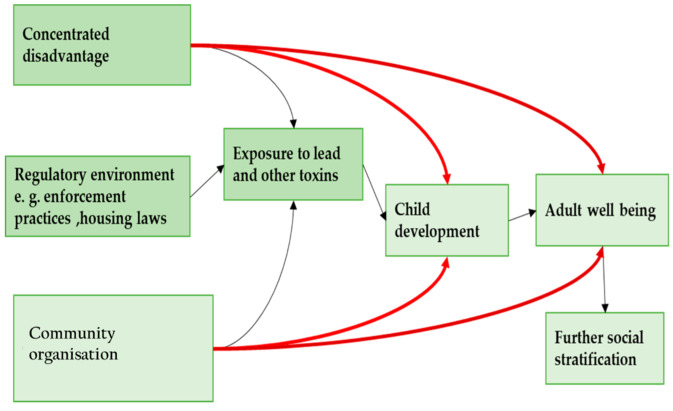
A model of inequality, environmental toxicity and well-being over the life course.

Previous studies on blood lead levels in the vicinity of a lead smelter indicate that lead contamination is spatially variable and depends mainly on proximity to the source of the emission. According to Etiang’ et al. [10], a child case study near the Owino Uhuru lead smelter, Mombasa, Kenya had a significantly higher Blood Lead Level (BLL) of 7.4 μg/dL compared to 3.7 μg/dL for a settlement in Bangladesh (*p* < 0.05) 2 km from the factory. Models of the interaction of lead sources and the immediate environment show that children are more susceptible to extreme levels of exposure to lead, as presented in Figure 5 [19]. 

Similarly, BLLs were found to be higher among 2nd- and 3rd-grade school children living in Jakarta, Indonesia near a highway or major intersection than those of children who lived near a street with little or no traffic [20,21].

A similar study near an lead–acid battery recycling operation in Kenya’s Nairobi slums found elevated levels of lead in soil and dust samples in vulnerable communities. Almost all children living in slums showed levels above 34 μg/dL [22]. Regarding this study, it is highly likely that communities within 900 m, such as the Makongeni, Mukuru and Mbotela slums, are exposed to lead contamination. Further investigation in relation to soils or dust with measurement samples of blood lead levels among the nearby population are strongly recommended. 

## 4. Conclusions

The result of this study confirms the need for developing countries to continuously monitor and control levels of lead concentrations to ensure a high-quality environment for surrounding communities.

Lead in air was found as the major vector of contamination of the surrounding environment, as well as deposits on plant leaves and potentially on other surfaces. Unit operation sections Mac-Parter and Super 85 were led the generation of lead-filled dust. Modernisation of these sections with lead-extracting equipment is recommended in order to extract dust and prevent Pb particles being released into the air.

The Pb concentration in wastewater indicated the need to consider further or alternative treatment, because the use of NaCO_3_ reagents turned out to be ineffective and could only achieve 9.89 ± 1.32% treatment efficiency. Exploration of additional efficient wastewater treatment methods is therefore necessary. While notable safety measures were observed and integrated in the operations of the company, frequent checks and review of the same safety procedures are indispensable. Ensuring good working conditions is advisable to prevent lead-filled environments. This research was conducted as a proactive and environmental monitoring approach and provided data for decision making. While this study paid particular attention to airborne lead contamination in AMB’s work area, further administrative and scientific steps should be extended to soil Pb monitoring in a specific area around the factory. This step is crucial to evaluate the long-term environmental impact of the battery company, in particular to assess the risk of groundwater contamination from lead migration with infiltrated water, as well as potential wastewater leaks from a defective sewage network. Such action will enable a more comprehensive health risk assessment, not only for factory employees, but also for the local community, notably exposed to lead poisoning.

## Figures and Tables

**Table 1:** Limits of selected heavy metals (ppm) in drinking water, wastewater, soils and vegetables as recommended by WHO and Kenya (NEMA and KEBS) (arranged from [13]).

Type of Sample	Lead Concentration Limits (ppm)	
	WHO	Kenya (NEMA and KEBS)
Drinking water	0.01	0.05
Wastewater (effluents)	0.01	0.01
Soils (for agriculture)	0.1	1
Plant leaves (vegetables)	0.1–0.3	0.3

## Data Availability

The data presented in this study are available in supplementary material.

## Supplementary Materials

The following supporting information can be downloaded at: https://www.mdpi.com/article/10.3390/ijerph19095195/s1, Table S1: The results of measurements of Lead (Pb) concentration in Air in ppm within the Associated Battery Manufacturing (East Africa). Table S2: The result of measurements of Lead (Pb) concentration in factory wastewater within the Associated Battery Manufacturing (East Africa). Table S3: The measurements of Lead (Pb) concentration in the plant extract in ppm within the Associated Battery Manufacturing (East Africa).
