# Supplementary material for: Monitoring Lead Concentration in the Surrounding Environmental Components of a Lead Battery Company: Plants, Air and Effluents—Case Study, Kenya
Source: Int J Environ Res Public Health. 2022 Apr 25;19(9):5195. doi: 10.3390/ijerph19095195 (PMC9103765; doi:10.3390/ijerph19095195)
Supplement: Supplementary file 1 [file ijerph-19-05195-s001.zip › ijerph-1638859-supplementary.pdf]

Table S1. The results of measurements of Lead (Pb) concentration in Air in ppm within the Associated Battery Manufacturing (East Africa).

Unit operation sections	Before Sampling			After Sampling			Weight of Lead Sampled in grams	Calculations			
	Weight of cartridge	Weight of cartridge & filter	Weight of filter WtB	Weight of cartridge	Weight of cartridge & filter	Weight of filter WtA	(WtA – WtB)	Flow rate (Litre/Min)	Volume of air Sampled (Litres)	Lead (ppm)	Lead In Air (mg/m3)
MacParter A	0.3517	0.37231	0.02061	0.3517	0.37339	0.02169	0.00108	1.2	602.5	1.81	0.150207
MacParter B	0.33664	0.35774	0.0211	0.33664	0.35781	0.02117	0.00007	1.2	601.8	1.43	0.11881
Super 85 A	0.35173	0.37222	0.02049	0.35173	0.3727	0.02093	0.00044	1.2	568	1.23	0.108275
Super 85 B	0.33666	0.35704	0.02038	0.33666	0.3579	0.02124	0.00086	1.2	567.9	1.03	0.090685
Pasting A(HOPPER)	0.35176	0.37243	0.02067	0.35176	0.373	0.02126	0.00059	1.2	590.2	0.7	0.059302
Pasting B (PASTE MIXING)	0.35182	0.37241	0.02059	0.35182	0.373	0.02113	0.00054	1.2	600.2	0.64	0.053316
Pasting A(STACKING)	0.33673	0.35728	0.02055	0.33673	0.3579	0.02112	0.00057	1.2	601.8	0.0055	0.000457

Table S2. The result of measurements of Lead (Pb) concentration in factory wastewater within the Associated Battery Manufacturing (East Africa).

Sample number	Before treatment in (ppm)			After treatment (ppm)		
	Run 1	Run 2	Average	Run 1	Run 2	Average
**1**	0.3255	0.3265	0.326	0.2874	0.289	0.2882
**2**	0.3125	0.3145	0.3155	0.283	0.2912	0.2871
**3**	0.3277	0.3277	0.3237	0.28	0.296	0.288
**4**	0.3187	0.3227	0.3207	0.29	0.2932	0.2916
**5**	0.3235	0.318	0.3185	0.289	0.2922	0.2906

Table S3. The measurements of Lead (Pb) concentration in the plant extract in ppm within the Associated Battery Manufacturing (East Africa).

Sample number	Sampling point	Run 1 (ppm)	Run 2 (ppm)	Average (ppm)
1	Area around local WWTP	1.2387	1.2389	1.2388
2	Distribution section	1.1835	1.1833	1.1834
3	Area around Casting	1.1945	1.2051	1.1998
4	Area around local WWTP	1.2231	1.2273	1.2252
5	Area around local WWTP	1.222	1.2254	1.2227
